# Prevalence and associated factors of depressive and anxiety symptoms among Chinese secondary school students

**DOI:** 10.1186/s12888-023-05068-1

**Published:** 2023-08-10

**Authors:** Zhangming Chen, Silan Ren, Ruini He, Yudiao Liang, Youguo Tan, Yi Liu, Fanglan Wang, Xu Shao, Shanshan Chen, Yanhui Liao, Ying He, Jin-guang Li, Xiaogang Chen, Jinsong Tang

**Affiliations:** 1 Department of Psychiatry, National Clinical Research Center for Mental Disorders, The Second Xiangya Hospital of Central South University, Changsha, Hunan China; 2 Department of Nursing, Sichuan Vocational College of Health and Rehabilitation, Zigong, Sichuan China; 3 Department of Psychiatry, Zigong Mental Health Center, Zigong, Sichuan China; 4 Department of Psychiatry, Sir Run Run Shaw Hospital, Zhejiang University School of Medicine, Hangzhou, Zhejiang China

**Keywords:** Adolescents, Mental health, Peer bullying, Internet gaming disorder

## Abstract

**Background:**

Depressive and anxiety symptoms affect about one-fourth of Chinese secondary school students. However, the prevalence and correlates of mental distress among secondary school students from Western China remain largely unexplored. This study aimed to examine the prevalence and associations of depressive and anxiety symptoms with demographic, family, school, life, and behavior factors in a large, representative sample of secondary school students in Zigong, a city in Western China.

**Methods:**

Secondary school students were recruited using cluster sampling. The 9-item Patient Health Questionnaire, the 7-item Generalized Anxiety Disorder Questionnaire, Multidimensional Peer-Victimization Scale, the Pittsburgh Sleep Quality Index, and Nine-Item Internet Gaming Disorder Scale-Short Form were used. Descriptive statistic was used to describe the sociodemographic characteristics of participants. The clustering effect was adjusted by the “survey” package of R to calculate weighted prevalence. Univariate and multivariate logistic regression were used to explore associated factors of depression and anxiety, respectively.

**Results:**

A total of 63,205 participants were involved, in which the weighted prevalence of depression in all subjects was 23.0% (*95% CI*: 19.6- 27.0%), and the weighted prevalence of anxiety was 13.9% (*95% CI*: 11.2- 17.0%). Logistic regression results showed girls, being single-child, non-nuclear family, peer bullying, sleep disturbance, and internet gaming disorder symptoms were positively associated with depressive and anxiety symptoms.

**Conclusion:**

Depressive and anxiety symptoms were prevalent among secondary school students in Western China. Our results can guide policy strategies for the assessment, prevention, and intervention of psychological status among Chinese secondary school students.

## Background

Based on the World Health Organization report [1], one-seventh of adolescents aged 10–19 years old experience a mental disorder, and depression and anxiety are the primary causes of disease and disability among this age group. During adolescence, all kinds of physical, mental, and interpersonal demands are required. When teens do not complete a specific requirement, worry and anxiety arise, leading to mental disorders like depression and anxiety. For example, depression increased from 2% in beginning puberty to around 18% in late puberty [2]. Based on a meta-analysis of the Chinese adolescent sample, the prevalence rate of depression was 24.3% [3], and rate of anxiety was 24.0% [4]. In response to these concerns, the Chinese government has introduced the Healthy China Action (2019-30) plan, which aims to enhance the mental well-being of Chinese adolescents. The plan emphasizes the establishment of a comprehensive mental health service network encompassing schools, communities, families, media, and medical institutions [5]. Specifically, schools are required to develop psychological service platforms or rely on school doctors to provide mental health support to students in distress.

Examining risk factors for depressive and anxiety symptoms in adolescents holds significant importance, as it enables parents, schools, and policymakers to identify at-risk individuals and provide timely interventions. A series of demographic, family, school, life, and behavior factors were found to be associated with mental distress among adolescents. For example, female gender was a stable predictor of depression and anxiety [6]. The higher grade may have a larger rate of depression and anxiety [6]. Although urban residence was an influencing factor of anxiety, its impact was controversial [7, 8], and findings of depression were likewise [9–11]. As to family factors, non-single child and non-intact families predicted stronger probability of persistent depression [12]. Children from single-parent families were more possible to suffer from depression [13] and anxiety [14]. Left-behind children experienced more depression and anxiety than non-left-behind [15]. Studies revealed that bullying victimization and physical abuse were associated with depressive and anxiety among adolescents [16]. Regarding life factors, sleep structure undergoes a dramatic change during adolescence, and teenagers are susceptible to sleep problems due to delayed biological circadian [17]. Sleep disturbance among adolescents was commonly found to be associated with depression [18] or anxiety [19]. As to behavioral factors, with the popularization of digital devices, students nowadays can easily get access to internet use, and online gaming can become addictive to adolescents [20]. Studies showed that adolescents with internet gaming disorder (IGD) had more severe depression and anxiety symptoms [21].

Despite extensive research, several knowledge gaps persist. Firstly, prior studies have highlighted significant regional disparities in the prevalence of mental distress among Chinese adolescents, attributed to economic and living conditions, with Western China recording higher rates [22]. However, focused research on the prevalence and factors associated with depressive and anxiety symptoms in Western Chinese adolescents is scarce; only 9 out of 51 publications in a meta-analysis on Chinese adolescents’ depression involved Western China [3]. Second, findings regarding the association of mental distress with demographic factors such as sex (boys versus girls) and study phase (middle school versus high school) have been inconsistent [3]. Third, previous studies have often utilized narrow measurement instruments that fail to comprehensively capture the impact of school, life, and behavioral factors on mental distress. For example, peer bullying, verbal or physical, exists in various forms but was not differentiated in previous studies. Simple questions were asked about sleep hours or internet gaming durations. Dimensions of sleep and degree of IGD are in lack of investigation. Taken together, a further large-scale study on the prevalence and associated factors of depressive and anxiety symptoms in adolescents from Western China is still warranted.

The present study aimed to: (1) assess the prevalence of depressive and anxiety symptoms in a large, representative sample of secondary schools in Zigong, a city in Western China; and (2) comprehensively evaluate the association of depressive and anxiety symptoms with demographic (sex, age, study phase, residence), family (single-child, left-behind children, family type), school bullying, sleep disturbance, and IGD symptoms.

## Methods

### Study design and participants

A school-based cross-sectional survey was conducted from September to December 2020 in Zigong City, situated in the southern part of Sichuan, China. At this time, Zigong was experiencing a remission phase of the COVID-19 pandemic. The school lockdown policy was ended and students went back to campus on April 2020. Through a cluster sampling approach, two districts and one county were selected from four districts and two counties in Zigong. The study encompassed all secondary schools (middle and high school) within these areas.

Investigators were meticulously trained in the study protocol and data collection standards to ensure the quality of the survey. Students completed an electronic questionnaire in the school computer rooms. Prior to the survey, both students and their parents were comprehensively informed about the survey’s purpose, procedures, and measurements, and provided their consent. A total of 63,487 junior high and senior high school students finished the investigation, and 282 were excluded due to missing data for critical items, leaving 63,205 subjects for the study, with an effective response proportion of 99.6%. The protocol was approved by the Ethics Committee of Zigong Mental Health Center [No. 2020-8-01].

### Measurement

A self-report questionnaire was applied in this study. Data were collected using a self-developed questionnaire. The questionnaire contained several parts: Socio-demographic information, including age, gender, study phase (middle school/ high school), residence (urban/rural), family type (non-nuclear family/ nuclear family), single-child (yes/ no), and left-behind children (yes/ no), depressive symptom, anxiety symptom, peer bullying, sleep disturbance, and internet gaming disorder (IGD). Participants were requested to report whether they belonged to a nuclear family or a non-nuclear family (single-parent family or reconstituted family). Left-behind children refer to children who are separated from one or both of their parents due to migration of their parents for work or other reasons.

Anxiety symptom was measured via the 7-item Generalized Anxiety Disorder Questionnaire (GAD-7) [23]. The scale contains 7 items, and each item is rated from 0 to 3, yielding a total score from 0 to 21. In this study, a total score of ≥ 10 represents positive for anxiety. The Chinese version of GAD-7 has demonstrated reliability and validity in the Chinese adolescents [24].

The 9-item Patient Health Questionnaire (PHQ-9) [25] was applied to assess depressive symptoms. The response option on each item ranges from “not at all” (0 points) to “nearly every day” (3 points), yielding a total score from 0 to 27. A score of ≥ 10 represents positive for depression. The PHQ-9 has been validated and widely used across different Chinese samples [26–29].

The multidimensional Peer-Victimization Scale (MPVS) measures peer bullying suitable for adolescents aged 11–16 [30, 31]. It has 16 items, each asking individuals how often they experienced each event over the past year, with response options ranging from 0 (not at all), 1 (once), to 2 (more than once). This instrument has four factors (four items each), namely physical victimization, verbal victimization, social manipulation, and property attacks. In the current analysis, factor score ≥ 1 represents the experience of respective subtypes of peer bullying.

Sleep disturbance was measured by the Pittsburgh Sleep Quality Index (PSQI) [32]. It has seven factors, rated from 0 to 3, yielding a global score from 0 to 21. A higher score indicates worse sleeping quality. A global score > 5 indicates ‘poor sleep’ and was considered positive for sleep disturbance in this study. This questionnaire is a structure-validated and globally recognized instrument with good reliability and validity [33].

IGD symptoms were assessed through the Nine-Item Internet Gaming Disorder Scale-Short Form (IGDS9-SF), which was developed by Pontes, Halley, M., Griffiths, Mark and D. [34]. Items are rated by a five-point Likert-type scale ranging from 1 (never) to 5 (very often), yielding a total score from 9 to 45. This instrument has been introduced to the Chinese samples, and a total score ≥ 32 was considered positive for Internet gaming addiction in the Chinese samples [35].

### Statistical analysis

Descriptive statistics were employed to summarize participant characteristics. Based on PHQ-9 and GAD-7 scores, participants were categorized into four groups: non-mental distress, anxiety symptoms only, depressive symptoms only, and both depressive and anxiety symptoms. Given the high co-occurrence of depressive and anxiety symptoms among our participants, a further classification was made into the non-distress group and distress group (participants displaying either depressive or anxiety symptoms).

Chi-square tests and t-tests were used to examine differences between the distress and non-distress groups in demographics, family-related information, sleep disturbance, school bullying, and IGD symptoms. Considering the recruitment method of the participants and the design of the study involved cluster sampling, the “survey” package of R was applied to get weighted prevalence based on cluster. A multivariate logistic regression model was applied to identify factors independently associated with mental distress. Variables that were statistically significant in univariate analysis were included. Consistent with a previous study [36], adjusted odds ratios (ORs) were used to evaluate effect size, with ORs < 1.5, between 1.5 and 4, and > 4 indicating weak, moderate, and strong associations, respectively.

We further performed a subgroup analysis to examine potential differences in the factors associated with mental distress between middle school and high school students.

Statistics were performed using R software (Version 3.3.3, The R Foundation for Statistical Computing, Vienna, Austria). The “survey” package was used to adjust the clustering effect. Given the very large sample size [36], the significance level was set at less than 0.001 (two-tailed).

## Results

### Major characteristics of the participants

A total of 63,205 subjects were included in the analysis, and their major characteristics are shown in Table 1. The mean age of all the participants was 14.3 years old, and 49.36% were boys. Junior high school students accounted for 68.62%. Among all the study subjects, 22.00% were single-child, and 35.13% were left-behind children. As for peer bullying, verbal victimization was reported the highest form (61%), followed by property attacks (59%), social manipulation (45%), and physical victimization (29%), respectively. Of all the participants, 30% reported sleep disturbance, and 2.9% were qualified as IGD.

**Table 1:** Sample Characteristics

Characteristic	Distress			p-value^2^
	Overall, N = 63,205^1^	Without distress, N = 47,107^1^	With distress, N = 16,098^1^	
**Gender**				< 0.001
Girl	32,007 (51%)	22,676 (48%)	9,331 (58%)	
Boy	31,198 (49%)	24,431 (52%)	6,767 (42%)	
**Study phase**				0.007
High school	19,832 (31%)	14,644 (31%)	5,188 (32%)	
Middle school	43,373 (69%)	32,463 (69%)	10,910 (68%)	
**Age, years**	14.33 (1.65)	14.32 (1.66)	14.37 (1.61)	< 0.001
**Residence**				< 0.001
Country	42, 059 (67%)	31, 640 (67%)	10,419 (65%)	
Urban	21,146 (33%)	15,467 (33%)	5,679 (35%)	
**Single child, yes**	13,904 (22%)	10,132 (22%)	3,772 (23%)	< 0.001
**Left-behind children, yes**	22,202 (35%)	16,366 (35%)	5,836 (36%)	< 0.001
**Family type**				< 0.001
Nuclear family	50,414 (80%)	38,425 (82%)	11,989 (74%)	
Non-nuclear family	12, 791(20%)	8, 682 (18%)	4, 109 (26%)	
**IGD symptoms, yes**	1,813 (2.9%)	474 (1.0%)	1,339 (8.3%)	< 0.001
**Sleep Disturbance, yes**	18,647 (30%)	7,946 (17%)	10,701 (66%)	< 0.001
**Physical victimization**	18,548 (29%)	10,812 (23%)	7,736 (48%)	< 0.001
**Verbal victimization**	38,660 (61%)	25,234 (54%)	13,426 (83%)	< 0.001
**Social manipulation**	28,544 (45%)	16,982 (36%)	11,562 (72%)	< 0.001
**Property attacks**	37,259 (59%)	24,369 (52%)	12,890 (80%)	< 0.001

^1^n (%); Mean (SD)

^2^Pearson’s Chi-squared test; Welch Two Sample t-test

### Weighted prevalence of depression and anxiety symptoms

The weighted prevalence of depressive and anxiety symptoms was 23.0% (*95% CI*: 19.6- 27.0%) and 13.9% (*95% CI*: 11.2- 17.0%), respectively. 11.5% (*95% CI*: 9.0- 15.0%) of secondary school students suffered from both depressive and anxiety symptoms. Approximately one-fourth of the participants (25.5%, *95% CI*: 21.8- 30.0%) displayed either depressive or anxiety symptoms and were classified into the distress group.

Compared to non-distress group, adolescents with mental distress were older (14.37 vs. 14.32). They were more likely to be girls (58% vs. 48%), single-child (23% vs. 22%), and left-behind children (36% vs. 35%). They were more likely to live in the urban (35% vs. 33%) and belong to a non-nuclear family (26% vs. 18%) (all p < 0.001). Notably, adolescents with mental distress were at a much higher risk for IGD symptoms (8.3% vs. 1.0%), sleep disturbance (66% vs. 17%), physical victimization (48% vs. 23%), verbal victimization (83% vs. 54%), social manipulation (72% vs. 36%), and property attacks (80% vs. 52%) (all p < 0.001).

### Associated factors of depression and anxiety symptoms

We performed multiple regression models to identify the independent factors associated with mental distress (Table 2). The following variables were selected in the regression model: gender, residence, age, being single-child, being left-behind children, family type, school bullying experience, sleep disturbance, and IGD symptoms. The model suggested that girls (OR: 1.55, 95% CI: 1.49–1.62, p < 0.001), belonging to a non-nuclear family (OR: 1.31, 95% CI: 1.25–1.38, p < 0.001), being single-child (OR, 1.06, 95%CI, 1.01–1.12, p = 0.025), living in urban (OR, 1.11; 95%CI, 1.06–1.17, p < 0.001), physical victimization (OR: 1.51, 95% CI: 1.44–1.59, p < 0.001), verbal victimization (OR: 1.70, 95% CI: 1.60–1.80, p < 0.001), social manipulation (OR: 1.97, 95% CI: 1.87–2.08, p < 0.001), property attacks (OR: 1.25, 95% CI: 1.18–1.32, p < 0.001), sleep disturbance (OR: 6.99, 95% CI: 6.69–7.30, p < 0.001), and IGD symptoms (OR: 5.00, 95% CI: 4.42–5.66, p < 0.001) was independently associated with mental problems.

**Table 2:** Univariate and multivariate analysis of influencing factors for mental distress in secondary school students (N = 63, 205)

Variables		Without distress	With distress	OR (univariable)	OR (multivariable)
Gender	Boy	24,431 (78.3)	6767 (21.7)	-	-
	Girl	22,676 (70.8)	9331 (29.2)	**1.49 (1.43–1.54, p < 0.001)**	**1.55 (1.49–1.62, p < 0.001)**
School	Middle school students	32,463 (74.8)	10,910 (25.2)	-	-
	High school students	14,644 (73.8)	5188 (26.2)	1.05 (1.01–1.10, p = 0.007)	-
Age	Mean (SD)	14.3 (1.7)	14.4 (1.6)	**1.02 (1.01–1.03, p < 0.001)**	0.99 (0.98-1.00, p = 0.148)
Residence	Country	31,640 (75.2)	10,419 (24.8)	**-**	-
	Urban	15,467 (73.1)	5679 (26.9)	**1.12 (1.07–1.16, p < 0.001)**	**1.11 (1.06–1.17, p < 0.001)**
Single-child	No	36,975 (75.0)	12,326 (25.0)	**-**	-
	Yes	10,132 (72.9)	3772 (27.1)	**1.12 (1.07–1.17, p < 0.001)**	**1.06 (1.01–1.12, p = 0.025)**
Left-behind child	No	30,741 (75.0)	10,262 (25.0)	**-**	-
	Yes	16,366 (73.7)	5836 (26.3)	**1.07 (1.03–1.11, p = 0.001)**	1.02 (0.97–1.07, p = 0.457)
Family type	Nuclear	38,425 (76.2)	11,989 (23.8)	**-**	-
	Non-nuclear	8682 (67.9)	4109 (32.1)	**1.52 (1.45–1.58, p < 0.001)**	**1.31 (1.25–1.38, p < 0.001)**
IGD	No	46,633 (76.0)	14,759 (24.0)	**-**	-
	Yes	474 (26.1)	1339 (73.9)	**8.93 (8.03–9.94, p < 0.001)**	**5.00 (4.42–5.66, p < 0.001)**
Sleep Disturbance	No	39,161 (87.9)	5397 (12.1)	**-**	**-**
	Yes	7946 (42.6)	10,701 (57.4)	**9.77 (9.38–10.18, p < 0.001)**	**6.99 (6.69–7.30, p < 0.001)**
Physical victimization	No	36,295 (81.3)	8362 (18.7)	**-**	**-**
	Yes	10,812 (58.3)	7736 (41.7)	**3.11 (2.99–3.22, p < 0.001)**	**1.51 (1.44–1.59, p < 0.001)**
Verbal victimization	No	21,873 (89.1)	2672 (10.9)	**-**	**-**
	Yes	25,234 (65.3)	13,426 (34.7)	**4.36 (4.16–4.56, p < 0.001)**	**1.70 (1.60–1.80, p < 0.001)**
Social manipulation	No	30,125 (86.9)	4536 (13.1)	**-**	**-**
	Yes	16,982 (59.5)	11,562 (40.5)	**4.52 (4.35–4.70, p < 0.001)**	**1.97 (1.87–2.08, p < 0.001)**
Property attacks	No	22,738 (87.6)	3208 (12.4)	**-**	**-**
	Yes	24,369 (65.4)	12,890 (34.6)	**3.75 (3.59–3.91, p < 0.001)**	**1.25 (1.18–1.33, p < 0.001)**

A subsequent subgroup analysis was conducted to investigate potential differences in factors associated with mental distress between middle school and high school students (Tables 3 and 4). The regression model revealed that school bullying victimization, IGD symptoms, sleep disturbance, family types, and being female consistently showed positive associations with mental problems in both middle school and high school students. However, age (OR, 1.06, 95%CI: 1.03–1.09, p < 0.001) and being a single-child (OR1.10, 95%CI: 1.03–1.17, p = 0.005) were only associated with mental distress in middle school students, while living in urban (OR, 1.24, 95%CI, 1.15–1.34, p < 0.001) was only associated with mental distress in high school students.

**Table 3:** Univariate and multivariate analysis of influencing factors for mental distress in middle school students

Variables		Without distress	With distress	OR (univariable)	OR (multivariable)
Gender	Boy	17,503 (78.4)	4819 (21.6)	-	-
	Girl	14,960 (71.1)	6091 (28.9)	**1.48 (1.42–1.54, p < 0.001)**	**1.56 (1.48–1.65, p < 0.001)**
Age	Mean (SD)	13.4 (1.0)	13.5 (1.0)	**1.07 (1.05–1.09, p < 0.001)**	**1.06 (1.03–1.09, p < 0.001)**
Residence	Country	22,468 (75.2)	7428 (24.8)	-	-
	Urban	9995 (74.2)	3482 (25.8)	1.05 (1.01–1.10, p = 0.028)	-
Single-child	No	25,708 (75.3)	8411 (24.7)	-	-
	Yes	6755 (73.0)	2499 (27.0)	**1.13 (1.07–1.19, p < 0.001)**	**1.10 (1.03–1.17, p = 0.005)**
Left-behind child	No	21,269 (75.7)	6845 (24.3)	**-**	-
	Yes	11,194 (73.4)	4065 (26.6)	**1.13 (1.08–1.18, p < 0.001)**	1.02 (0.97–1.08, p = 0.411)
Family type	Nuclear	26,077 (76.8)	7894 (23.2)	**-**	-
	Non-nuclear	6386 (67.9)	3016 (32.1)	**1.56 (1.48–1.64, p < 0.001)**	**1.32 (1.24–1.40, p < 0.001)**
IGD	No	32,096 (76.4)	9890 (23.6)	**-**	-
	Yes	367 (26.5)	1020 (73.5)	**9.02 (8.00-10.20, p < 0.001)**	**4.92 (4.27–5.68, p < 0.001)**
Sleep Disturbance	No	28,158 (87.4)	4077 (12.6)	**-**	-
	Yes	4305 (38.7)	6833 (61.3)	**10.96 (10.43–11.53, p < 0.001)**	**7.29 (6.91–7.69, p < 0.001)**
Verbal victimization	No	14,411 (90.7)	1484 (9.3)	**-**	-
	Yes	18,052 (65.7)	9426 (34.3)	**5.07 (4.78–5.38, p < 0.001)**	**1.61 (1.51–1.71, p < 0.001)**
Physical victimization	No	24,210 (83.0)	4943 (17.0)	**-**	-
	Yes	8253 (58.0)	5967 (42.0)	**3.54 (3.38–3.71, p < 0.001)**	**1.74 (1.61–1.88, p < 0.001)**
Social manipulation	No	20,396 (88.2)	2733 (11.8)	**-**	-
	Yes	12,067 (59.6)	8177 (40.4)	**5.06 (4.82–5.31, p < 0.001)**	**2.04 (1.91–2.18, p < 0.001)**
Property attacks	No	15,502 (88.8)	1961 (11.2)	**-**	-
	Yes	16,961 (65.5)	8949 (34.5)	**4.17 (3.95–4.40, p < 0.001)**	**1.22 (1.13–1.31, p < 0.001)**

**Table 4:** Univariate and multivariate analysis of influencing factors for mental distress in high school students

Variables		Without distress	With distress	OR (univariable)	OR (multivariable)
Gender	Boy	6928 (78.1)	1948 (21.9)	-	-
	Girl	7716 (70.4)	3240 (29.6)	**1.49 (1.40–1.59, p < 0.001)**	**1.58 (1.47–1.71, p < 0.001)**
Age	Mean (SD)	16.3 (1.0)	16.2 (1.0)	**0.93 (0.90–0.96, p < 0.001)**	0.97 (0.93–1.01, p = 0.096)
Residence	Country	9172 (75.4)	2991 (24.6)	**-**	-
	Urban	5472 (71.4)	2197 (28.6)	**1.23 (1.15–1.31, p < 0.001)**	**1.24 (1.15–1.34, p < 0.001)**
Single-child	No	11,267 (74.2)	3915 (25.8)	-	-
	Yes	3377 (72.6)	1273 (27.4)	1.08 (1.01–1.17, p = 0.031)	-
Left-behind child	No	9472 (73.5)	3417 (26.5)	-	-
	Yes	5172 (74.5)	1771 (25.5)	0.95 (0.89–1.01, p = 0.125)	-
Family type	Nuclear	12,348 (75.1)	4095 (24.9)	-	-
	Non-nuclear	2296 (67.7)	1093 (32.3)	**1.44 (1.32–1.56, p < 0.001)**	**1.26 (1.15–1.39, p < 0.001)**
IGD	No	14,537 (74.9)	4869 (25.1)	**-**	-
	Yes	107 (25.1)	319 (74.9)	**8.90 (7.16–11.15, p < 0.001)**	**4.94 (3.85–6.39, p < 0.001)**
Sleep Disturbance	No	11,003 (89.3)	1320 (10.7)	**-**	-
	Yes	3641 (48.5)	3868 (51.5)	**8.86 (8.24–9.53, p < 0.001)**	**6.58 (6.10–7.10, p < 0.001)**
Physical victimization	No	12,085 (77.9)	3419 (22.1)	**-**	-
	Yes	2559 (59.1)	1769 (40.9)	**2.44 (2.27–2.62, p < 0.001)**	**1.25 (1.14–1.37, p < 0.001)**
Verbal victimization	No	7462 (86.3)	1188 (13.7)	**-**	-
	Yes	7182 (64.2)	4000 (35.8)	**3.50 (3.25–3.76, p < 0.001)**	**1.64 (1.49–1.81, p < 0.001)**
Social manipulation	No	9729 (84.4)	1803 (15.6)	**-**	-
	Yes	4915 (59.2)	3385 (40.8)	**3.72 (3.48–3.97, p < 0.001)**	**1.84 (1.69-2.00, p < 0.001)**
Property attacks	No	7236 (85.3)	1247 (14.7)	**-**	-
	Yes	7408 (65.3)	3941 (34.7)	**3.09 (2.87–3.32, p < 0.001)**	**1.29 (1.18–1.42, p < 0.001)**

## Discussion

Our study is, to our knowledge, the largest of its kind to examine the prevalence and correlates of depression and anxiety among secondary school students in Western China. The major findings included: (1) Depressive and anxiety symptoms affected 23% and 14% of the students, respectively. About one-fourth of the students experienced mental distress; (2) Mental distress was independently associated with sex, family type, single-child status, residence, school bullying, sleep disturbance, and IGD symptoms; and (3) The prevalence of mental distress did not differ significantly between middle school and high school students, but some demographic and family factors had different associations with mental distress in the two groups. Our findings underscore the urgent need for formal assessments and interventions for mental distress in Chinese secondary school students. Our results can inform the early detection and timely intervention for students at risk of mental distress.

### Prevalence of depression and anxiety

We found that approximately one-fourth of the secondary school students suffered from depressive and/or anxiety symptoms. This was slightly lower than the global prevalence of 30% among adolescents during the COVID-19 pandemic reported by a recent meta-analysis [37]. A possible explanation could be the timing of our study. We recruited adolescents in the remission phase of the pandemic, when they had returned to campus for about half a year.

Compared to previous studies in the non-pandemic period, the prevalence of depression (23%) was fairly close to that in western China (22.7-24.8%) but slightly higher than that in Northeast (14.5%) and Southern China (17.8%) [22, 38, 39]. It was also in line with a previous meta-analysis, which demonstrated 24.3% of the Chinese secondary school students suffered from depressive symptoms [3]. Similarly, the prevalence of anxiety symptoms also coincided with previous research among Chinese secondary students (13.7 to 24.5%) [40, 41]. Notably, the prevalence of depressive and anxiety symptoms was much higher than that in the Chinese general population (8.6% for depressive symptoms and 6% for anxiety symptoms) [42]. There might be a few possible explanations for the high risk of mental distress in Chinese secondary school students. First, the Chinese education system is very competitive, and secondary school students are under great pressure. Many studies have shown that academic problems and depression in Chinese adolescents are highly correlated [43]. Second, Chinese secondary school students suffer from lots of interpersonal stress, and their parents are more likely to have high expectations of them and less likely to be satisfied with their academic performance [3].

### Associated factors of depressive and anxiety symptoms

Most demographic and family factors exhibited a rather weak association with mental distress, with sex and family structure being notable exceptions. Consistent with prior national studies [44], we found girls are at a higher risk for mental distress. Both biological (e.g., higher cortisol levels in girls when facing stressful events) and social-cultural factors (e.g., social stressors and gender intensification) might play a role in the sex difference of mental distress [45]. Another associated factor for mental distress is belonging to a non-nuclear family, which was consistent with previous studies [46]. Lower social support and higher parental stress such as financial and emotional stresses might contribute to the higher risk for mental distress among adolescents in non-nuclear family. Interestingly, we found several demographic and family factors (i.e., being single-child, residence, and age) exhibited varied levels of association with mental distress among middle school and high school students. Further studies are warranted to determine the potential mechanism.

We found all subtypes of school bullying were positively associated with mental distress. Social manipulation seemed to be the most harmful form of school bullying, which doubled the risk of mental distress in adolescents. Previous studies have found that the rate of school bullying varies from 9.00 to 61.6% [47], of which 35% are victims of traditional school bullying and 15% are victims of cyber school bullying. One Chinese research including 1252 high school students showed that 42.43% of students reported school bullying [16]. The previously reported incidence of school bullying among Chinese teenagers and vocational school students varied from 9.4 to 30.4% [48–50]. This suggests that bullying is a common appearance in schools. The result of our study is coincident with a previous study that found a significant correlation between bullying victimization and depression among high school students in China [16]. Our study is consistent with the stress theory, which states that suffering from bullying and being unable to cope with it may lead to stress, influencing mental health, and resulting in emotions like fear, depression, and anger. Wherefore, schools should strengthen the prevention of school bullying and better the school atmosphere to help reduce the level of depression in students.

Similar to other studies, we found that sleep disturbances were associated with students’ depressive and anxiety symptoms. Lots of studies showed that sleep has a significant impact on both physical and psychological health [51]. Facing major crises, good sleep can play a key role in addressing negative impacts [51]. In turn, poor sleep may exacerbate negative impacts and result in more vulnerability to anxiety [52]. Therefore, it is important to take steps to improve sleep in adolescents.

We found that students with IGD symptoms were more likely to suffer from anxiety and depression symptoms. Previous studies have found that most gaming-addicted students had experienced stressful life events [53]. Furthermore, C Bonnaire and O Phan [54] reported that bad family relationships have a strong impact on the prevalence of adolescent gaming addiction. The relationship between internet gaming addiction and boredom tendencies, thrill-seeking, and schizophrenic character has been found in some studies, including non-clinical samples [55].

### Implications of the findings

Our study holds two significant implications. First, our study highlights the high prevalence of mental distress, school bullying, and sleep disturbance in secondary school students in Western China, which calls for regular screening. Second, our study comprehensively evaluated the associated factors of depressive and anxiety symptoms. Specifically, school bullying, sleep disturbance, and IGD symptoms were closely associated with mental distress. The findings could help parents, teachers, and policymakers identify students at risk and develop targeted interventions. For example, schools and parents should promote sleep hygiene education, create a conducive sleeping environment, and help set a regular sleep schedule for secondary school students. Regarding bullying, teachers and schools should consider implementing anti-bullying programs that educate students about the consequences of bullying and promote a culture of respect and inclusion. Also, early detection of school bullying is of importance.

### Strength and limitation

Our study has several strengths, such as being the largest study on depressive and anxiety symptoms among secondary school students in Western China and assessing a comprehensive range of demographic, family, life, and behavior factors. However, several limitations should be acknowledged. First, the cross-sectional study design could not establish the causal relationship. Second, the survey was conducted after the outbreak of the pandemic. Although the school lockdown policy was ended and adolescents returned to school in April 2020, the cross-sectional nature of our study made it hard to determine the impact of the pandemic on our results. Third, despite the large sample size, all participants were recruited from one single city in western China. The generalizability of our findings to other regions of China requires further studies to confirm. Forth, depressive and anxiety symptoms are associated with addiction problems such as smoking and alcohol. However, the present study only focused on IGD symptoms. Future research could benefit from a more comprehensive analysis encompassing various forms of addiction among adolescents.

## Conclusion

In summary, one-fourth of the secondary school students in Western China suffered from depressive and anxiety symptoms. Sex, family type, being single-child, residence, school bullying, sleep disturbance, and IGD symptoms were independently associated with mental distress. Parents, schools, and policymakers should be aware of the high prevalence of mental distress in secondary school students. Our findings can help identify students at risk and provide tailored interventions.

## Data Availability

The datasets used and/or analyzed during the current study are available from the corresponding author on reasonable request.

## Abbreviations

IGD: Internet gaming disorder
PHQ-9: 9-item Patient Health Questionnaire
GAD-7: 7-item Generalized Anxiety Disorder Questionnaire
PSQI: Pittsburgh Sleep Quality Index
MPVS: Multidimensional Peer-Victimization Scale
IGDS9-SF: Nine-Item Internet Gaming Disorder Scale-Short Form
